# SX-ELLA biodegradable stent for benign oesophageal strictures: a systematic review and proportion meta-analysis

**DOI:** 10.1007/s00464-022-09767-w

**Published:** 2022-12-08

**Authors:** Elisha Kailla, Fatema Rezai, Ange Kamille Kansci, Oluwadamilola Akande, James Gossage

**Affiliations:** 1 GKT School of Medical Education, King’s College London, London, UK; 2 University of Birmingham Medical School, Birmingham, UK; 3 Department of Upper Gastrointestinal Surgery, St Thomas’ Hospital, London, UK

**Keywords:** Benign oesophageal stricture, Oesophageal stenosis, Biodegradable stent, Absorbable implants, Health technology assessment, Adults

## Abstract

**Background:**

This systematic review aimed to analyse the use of the SX-ELLA biodegradable stent (BDS) for benign oesophageal strictures through the assessment of clinical and technical success, differences in pre- and post-BDS insertion dysphagia scores, rates of stent migration, and safety.

**Methods:**

A systematic review was reported according to PRISMA guidelines, with a prospectively registered protocol. The databases PubMed, Embase, SCOPUS, and ClinicalTrials.gov were searched up to March 2022. Studies assessing the use of the SX-ELLA BDS in adults with benign oesophageal strictures were included. A pooled data analysis was conducted to analyse the clinical and technical success associated with BDS use, rate of stent migration, and safety.

**Results:**

Of the 1509 articles identified, 16 studies treating 246 patients were eligible for inclusion. BDS was clinically successful in 41.9% of cases (95% CI = 35.7 – 48.1%), defined as those who experienced complete symptom resolution following BDS insertion. Technical success was achieved in 97.2% of patients (95% CI = 95.1 – 99.3%). A pooled analysis concluded a decrease in mean dysphagia score of 1.8 points (95% CI = 1.68 – 1.91) following BDS insertion. Re-intervention was required in 89 patients (36.2%, 95% CI = 30.2 – 42.2%), whilst stent migration occurred in 6.5% of patients (95% CI = 3.4 – 9.6%). A total of 37 major clinical complications related to BDS insertion were reported (15.0%, 95% CI = 10.5 – 19.5%).

**Conclusion:**

The pooled data analysis demonstrates the high technical and moderate clinical success of the SX-ELLA biodegradable stent, supporting its use for benign oesophageal strictures in adults. However, greater evidence is required for more robust conclusions to be made in terms of success when compared to alternative methods of intervention, such as endoscopic dilation.

**Supplementary Information:**

The online version contains supplementary material available at 10.1007/s00464-022-09767-w.

Benign oesophageal strictures (BOS) are characterised by narrowing of the oesophageal lumen, due to inflammation, fibrosis, or neoplasm [1]. The incidence of BOS is 1.1 per 10,000 person-years and increases with age [2]. Despite treatment, 30–60% of BOS will recur [1]. Symptoms of BOS include dysphagia most commonly, as well as odynophagia, nausea, vomiting, and globus sensation [1]. The chronic inability to swallow solids and/or liquids leads to malnutrition, unintentional weight loss, and psychological effects, such as depression. BOS can be further classified as complex or simple, with complex strictures being severely narrowed, > 2 cm long, with a tortuous and irregular structure [3].

Cases of BOS have various causes [1], including post-surgical BOS at anastomotic sites, with 22–50% of oesophagectomy patients experiencing stricture formation requiring repeated dilations. BOS is a common complication of radiotherapy being administered to the neck and thoracic area, with higher doses of radiation being associated with a greater risk of stricture formation. Peptic BOS are caused by chronic gastro-oesophageal reflux disease (GORD) and the incidence of such strictures is declining due to the success of proton-pump inhibitor (PPI) therapy. BOS can also result from the ingestion of corrosive substances, a cause more common in younger age groups. Caustic strictures are the most common type to behave in a refractory manner. Additionally, other causes exist such as iatrogenic BOS and BOS resulting from eosinophilic or infective oesophagitis.

Current British Society of Gastroenterology (BSG) guidelines specify the use of fully covered self-expandable metal stents (FC-SEMS) in BOS refractory to conventional treatment of balloon or bougie dilation [4]. Further BSG recommendations include biodegradable stents (BDS) as an adjunct to endoscopic dilation for the treatment of BOS. In addition to FC-SEMS and BDS oesophageal stents, partially covered self-expandable metal stents (PC-SEMS) and self-expandable plastic stents (SEPS) are available. The complications of SEMS are problematic, involving stent migration and hyperplastic tissue reaction, the latter specifically from PC-SEMS. Both of these complications require stent removal [5]. However, the use of novel devices such as the Stentfix OTSC system has aided in reducing SEMS stent migration rates [6]. Likewise, SEPS are prone to stent migration and demonstrate varying efficacy in the literature [7].

This systematic review aims to address the SX-ELLA biodegradable stent for benign oesophageal strictures.

## Methods

### Biodegradable stent types

The focus of this review is the SX-ELLA biodegradable stent. Developed in the Czech Republic, the SX-ELLA biodegradable stent is composed of woven monofilament polydioxanone (PDS). Currently, this stent is the only BDS available as previous designs such as the ‘AB Esophacoil’ have been unsuccessful in their clinical application [8]. Use of the SX-ELLA stent has been documented in the UK, Spain, Italy, Belgium, the Netherlands, and Turkey, as well as in Japan, Pakistan, and India. The SX-ELLA BDS has lengths of 60, 80, 100, and 115 mm and diameters of 18, 20, 23, and 25 mm, exerting a radial force for 4–5 weeks. The BDS is inserted endoscopically using a delivery system typically under fluoroscopic guidance. The radiopaque gold markers on the stent allow for visualisation in radiological studies. The biodegradable nature of the stent means that no removal of the BDS is required as the stent undergoes hydrolysis and dissolves at 11–12 weeks.

### Inclusion and exclusion criteria

Our search was based on the following inclusion criteria: patients 18 years and over, with benign oesophageal strictures causing dysphagia, receiving SX-ELLA BDS insertion. We focussed on only the SX-ELLA BDS stent as this is currently the only biodegradable stent used in clinical practice in the management of benign oesophageal strictures. Patients under the age of 18 or patients with strictures associated with an active malignancy or oesophageal motility condition such as achalasia cardia were excluded. All study designs addressing the role of SX-ELLA BDS in benign oesophageal strictures were included. Papers such as review articles, conference abstracts, editorials, and book chapters were also excluded.

### Literature search

Two independent investigators (EK and FR) conducted a literature search of the following databases: PubMed, EMBASE, and SCOPUS to identify all relevant papers published up to March 2022, with no language restrictions. The search terms ‘oesophageal stricture’, ‘benign’, and ‘stent’ were used, and the alternative spelling of ‘esophagus’ was also accounted for. The Boolean operators ‘AND’ and ‘OR’ were utilised to ensure all appropriate literature was found. The ClinicalTrials.gov database was searched for any current, ongoing trials utilising BDS to manage benign oesophageal strictures. We included papers that analysed the role of BDS in benign oesophageal strictures alone or in comparison to other interventions. The title, abstract, and full text of studies were screened to ensure they met the inclusion criteria. The reference lists of the full-text studies were also reviewed to identify any additional papers.

### Data extraction

Following the screening process, the two investigators compared search results, ensuring any discrepancies in the included and excluded studies were discussed and rectified. A standardised data extraction form was created to compile the study characteristics of the included papers. The form was completed by one investigator (EK) and reviewed by a second (FR) to ensure the accuracy of inputted data. The following information was noted: year of study, country, the total number of participants, number of participants with benign oesophageal strictures, mean age, gender, stricture pathogenesis, mean stricture length, stricture site, technical success, clinical success, episodes of stent migration, the incidence of re-intervention, and major clinical complications.

The stricture pathogenesis was classified as ‘post-surgical’, ‘radiation’, ‘caustic’, ‘peptic’, or ‘other’, whilst the stricture site was split into ‘upper’, ‘middle’, and ‘lower’ thirds of the oesophagus.

### Outcomes of interest

Two primary outcomes were specified: dysphagia relief and incidence of re-intervention. Dysphagia was measured through the 5-point Ogilvie Dysphagia scale [9]. Clinical success was defined as complete dysphagia resolution. Incidence of re-intervention included patients undergoing further procedures for stent repositioning, or insertion, and those requiring endoscopic dilation or conversion to surgical procedures such as oesophageal resection or bypass in the event of stricture recurrence.

Secondary outcomes included technical success, defined as the BDS being inserted into the correct site without any complications. Incidence of stent migration was also assessed as well as, major clinical complications, defined as those related to the insertion of the BDS requiring intervention.

### Risk of bias assessment

The bias of each study was assessed using the appropriate tool. For RCTs, the Cochrane Risk of Bias (RoB 2) tool [10] was used. The bias of non-randomised studies was evaluated using the Risk of Bias in Non-randomised Studies – of Interventions (ROBINS-I tool) [11], whilst case series were assessed through the Joanna Briggs Institute (JBI) bias tool [12].

### Statistical analysis

Analysis of pre- and post-BDS insertion dysphagia scores was performed through pooled mean calculations. Furthermore, the 95% confidence intervals were calculated for the appropriate outcomes assessed.

## Results

### Search results

**Fig. 1:**
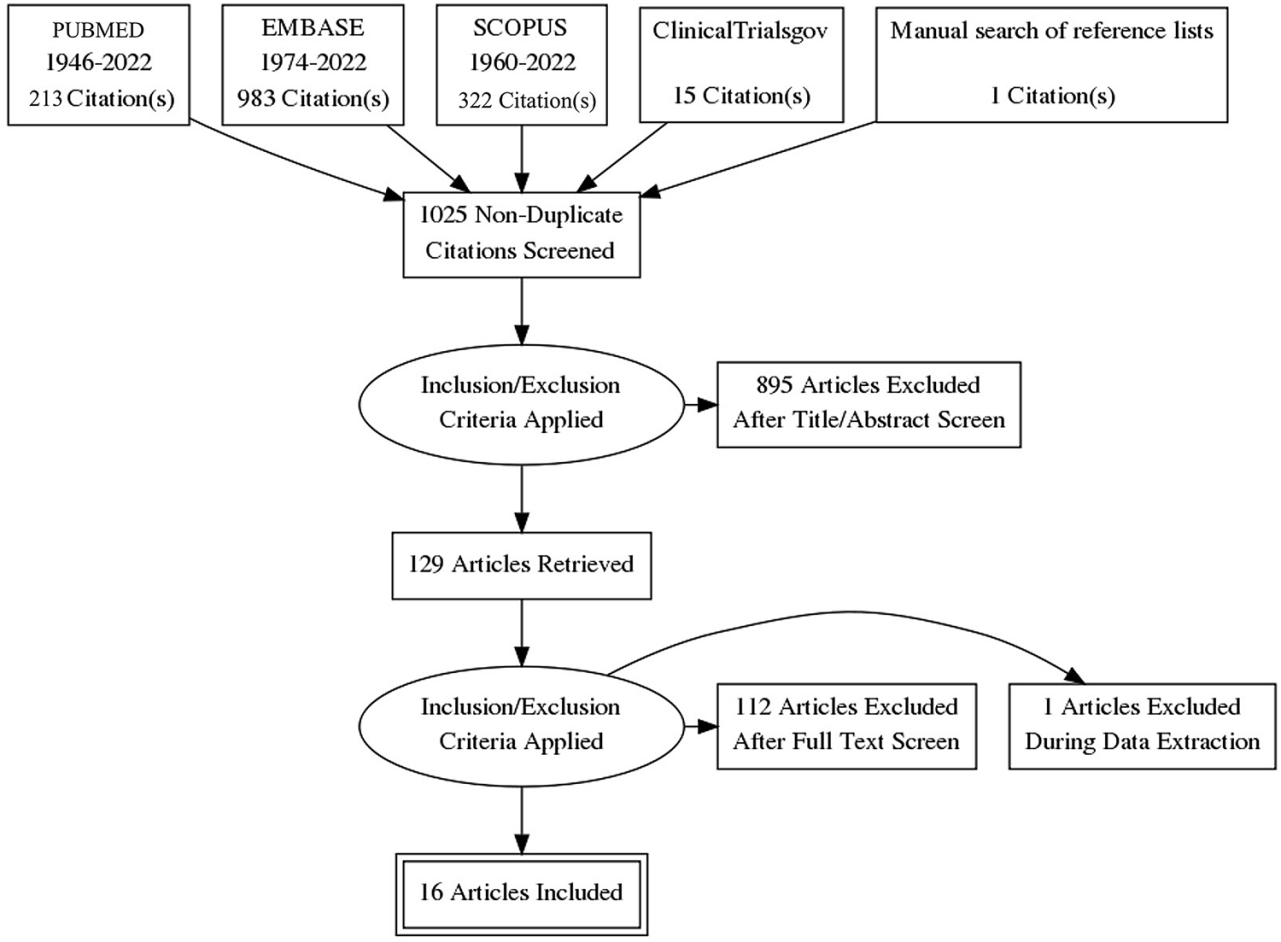
A Preferred Reporting Items for Systematic Reviews and Meta-Analysis (PRISMA) flow diagram

In total, 1509 studies were identified through database searches and one additional study was found through manual searching of reference lists. Following the removal of duplicates, 1025 studies underwent a title and abstract screen, leaving 129 full-text studies to be screened, 16 of which were eligible for inclusion. Reasons for exclusion include studies not matching the inclusion criteria, results for benign and malignant strictures being reported in tandem, and the use of biodegradable stents to treat other oesophageal pathologies such as leaks. A full PRISMA diagram of the screening process is shown in Fig. 1.

The pooled data of the studies equated to a total of 246 participants included in this review. Five of the included studies compared BDS to other methods of BOS management: fully covered SEMS, SEPS, and dilation (balloon and bougie), the remaining 11 assessed the role of BDS alone. Of the 16 studies included, there was one RCT, one pilot RCT, two prospective cohort studies, one retrospective cohort study, and 11 case series studies, seven of which were prospective and four retrospectives. The full study characteristics of the included studies are demonstrated in Table 1.

**Table 1:** Main characteristics of the included studies

Comparator studies						
Study author	Study design	Country	Comparator	Patients included (N)	Mean age (years)	Males N (%)
Canena et al. (2012)[13]	Prospective Cohort	Portugal	SEPS FC-SEMS	10	51	4 (40)
Dhar et al. (2014) [14]	Pilot RCT	UK	Balloon Dilation	9	63	8 (89)
Nogales et al. (2017) [15]	Retrospective Cohort	Spain	FC-SEMS	12	64	7 (58.3)
Van Boeckel et al. (2011) [16]	Prospective Cohort	Netherlands	SEPS	18	61	10 (56)
Walter et al. (2018) [17]	RCT	Netherlands	Dilation (balloon or bougie)	32	62	21 (65.5)

Non-comparator studies						
Study author	Study design	Country	Total patients (N)	Patients included (N)	Mean age (years)	Males N (%)
Griffiths et al. (2012) [18]	Prospective	UK	23	7	NR	NR
Hirdes et al. (2012) [19]	Prospective	Netherlands	28	28	58	15 (54)
Karakan et al. (2013) [20]	Prospective	Turkey	7	5	30	4 (80)
Kochhar et al. (2017) [21]	Retrospective	India	13	13	39	8 (62)
McCain et al. (2015) [22]	Retrospective	UK	29	18	71	14 (78)
Repici et al. (2010) [23]	Prospective	Italy, Netherlands	21	21	59	11 (52)
Saeed et al. (2018) [24]	Retrospective	Pakistan	17	5	51	7 (33.3)
Sigounas et al. (2016) [25]	Retrospective	UK	10	10	79	3 (30)
Van Hooft et al. (2011) [26]	Prospective	Netherlands	10	10	62	8 (80)
Yano et al. (2017) [27]	Prospective	Japan	18	18	71	15 (83.3)
Yano et al. (2022) [28]	Prospective	Japan	30	30	69	27 (90)
Overall (16 studies)			367	246	41	162 (65.9)

### Descriptive study analysis

The 16 studies included were conducted between 2010 and 2022, four of which were based in Asia, and the remaining studies were European. This systematic review analysed a total of 246 participants, with studies having a median sample size of 13 participants undergoing BDS insertion (ranging from 5 – 32 participants). The study’s participants had a mean age of 41 years old, 65.9% of whom were male.

Stricture pathogenesis of participants was classified as post-surgical in 37.6%, radiation in 10.4%, peptic in 13.7%, caustic in 11.6%, and other in 26.7% of cases. Further stricture characteristics include a mean stricture length of 3.03 cm, and differences in stricture site, with 29.6% of strictures occurring in the upper third of the oesophagus, 20.8% in the middle third, and a final 49.6% in the lower third. Full details of the stricture characteristics can be found in Table 2.

**Table 2:** Main characteristics of the benign oesophageal strictures as reported in the included studies

Study author	Stricture pathogenesis					Mean stricture length (cm)	Oesophageal stricture site		
	Post-surgical	Radiation	Peptic	Caustic	Other		Upper	Middle	Lower
	N (%)						N (%)		
Canena et al. (2012) [13]	6 (60)	–	3 (30)	1 (10)	–	2.9	–	–	10 (100)
Dhar et al. (2014) [14]	–	–	–	–	9 (100)	3.5	–	–	–
Griffiths et al. (2012) [18]	1 (20)	1 (20)	–	–	3 (60)	–	–	–	–
Hirdes et al. (2012) [19]	7 (25)	3 (11)	9 (32)	2 (7)	7 (25)	3.9	7 (25)	7 (25)	14 (50)
Karakan et al. (2013) [20]	–	–	–	5 (100)	–	5	1 (8)	9 (69)	3 (23)
Kochhar et al. (2017) [21]	–	–	–	13 (100)	–	4	–	3 (60)	2 (40)
McCain et al. (2015) [22]	2 (11)	–	–	–	16 (89)	–	5 (28)	3 (17)	10 (55)
Nogales et al. (2017) [15]	4 (33.3)	4 (33.3)	–	2 (16.7)	2 (16.7)	1.5	12 (100)	–	–
Repici et al. (2010) [23]	5 (24)	5 (24)	7 (33)	2 (9.5)	2 (9.5)	3	2 (9.5)	2 (9.5)	15 (81)
Saeed et al. (2018) [24]	4 (80)	1 (20)	–	–	–	–	–	–	–
Sigounas et al. (2016) [25]	1 (10)	2 (20)	7 (70)	–	–	4	–	2 (20)	8 (80)
Van Boeckel et al. (2011) [16]	5 (27)	2 (11)	6 (33)	2 (11)	3 (18)	4	10 (100)	–	–
Van Hooft et al. (2011) [26]	9 (90)	–	–	–	1 (10)	1	–	–	–
Walter et al. (2018) [17]	23 (71.9)	–	1 (3.1)	1 (3.1)	2 (21.9)	1	–	–	–
Yano et al. (2017) [27]	11 (61)	6 (33.3)	–	–	1 (5.7)	3.3	–	–	–
Yano et al. (2022) [28]	11 (36.6)	1 (3.4)	–	–	18 (60)	2.25	–	–	–
Overall	89 (37.6)	25 (10.4)	33 (13.7)	28 (11.6)	64 (26.7)	3.03	37 (29.6)	26 (20.8)	62 (49.6)

Regarding the diameter of SX-ELLA BDS used, two studies used 18-mm BDS for all participants and seven studies used 25-mm stents for all participants. One study used a diameter of 20 mm universally, whilst another used 23 mm. A single study had a mix of 23-mm and 25-mm stent diameters and a final study used 18-, 20-, and 23-mm stents. Three of the sixteen studies did not disclose the diameter of the stent used. Assessment of stent length was possible in only five studies, with lengths of 60, 80, 100, and 115 mm used.

### Risk of bias

All included studies were assessed using the appropriate bias tool. The two RCTs were assessed using the RoB 2 tool. Both RCTs had an overall bias judgement of ‘some concern’. The three non-randomised studies were assessed using the ROBINS-I tool, with the two studies scoring an overall moderate risk of bias and the third a serious risk of bias. The JBI tool was used for the 11 case series, all of which scored above five on the JBI scale, so all were included in the final data analysis. Full details of the bias tools and judgement can be found in the supplementary material for this review.

### Primary outcomes

Pre- and post-BDS dysphagia scores were reported in 10 studies, all of which are scored using the Ogilvie Dysphagia scale, as shown in Table 3. The pooled mean pre-BDS dysphagia score was 2.7, following BDS insertion this pooled mean was reduced to 0.9. Therefore, following SX-ELLA BDS insertion, the mean dysphagia score was reduced by 1.8 (95% CI = 1.68–1.91).

**Table 3:** Mean dysphagia scores as reported in the included studies, measured using the Ogilvie Dysphagia scale [8]

Pooled mean dysphagia score*		
Pre-BDS insertion	Post-BDS insertion	Mean difference
2.7	0.9	1.8
*Dysphagia scores measured through the 5-point Ogilvie Dysphagia scale: 0 = No dysphagia, 1 = Moderate passage: able to eat some solid foods, 2 = Poor passage: able to eat semi-solid foods, 3 = Very poor passage: able to swallow liquids only, 4 = No passage: unable to swallow any substance		

Re-intervention following BDS insertion was reported in 14 studies with a total of 89 re-interventions made (36.2%, 95% CI = 30.2 – 42.2%) due to stricture recurrence post-BDS hydrolysis and the re-onset of symptoms, notably dysphagia. The most common method of re-intervention was monthly endoscopic dilation, used 62 (69.7%) times. Second was endoscopic stenting, used in 24 (26.9%) instances. Both BDS and SEMS were utilised for re-intervention. The remaining three cases (3.4%) involved surgical re-intervention. The median time to re-intervention following the re-onset of dysphagia was reported to be between 100 and 260 days post-BDS insertion.

Clinical success occurred in 41.9% (95% CI = 35.7 – 48.1%) of participants, representing those who experienced complete resolution of symptoms following BDS insertion, without the need for any further intervention at the end of follow-up. The median duration participants spent dysphagia free varied greatly between the studies, from 2 to 18.5 months, post-BDS insertion.

### Secondary outcomes

This systematic review assessed three secondary outcomes: the technical success of BDS insertion, the incidence of BDS stent migration, and safety analysed through the study of major complications related to BDS insertion.

Technical success of BDS insertion occurred in 97.2% (95% CI = 95.1 – 99.3%) of participants, see Table 4. Unsuccessful stent insertion occurred in the seven cases. Three of these included a functional issue with the delivery system used for BDS insertion locking. In one patient the insertion of the BDS was abandoned due to the stricture being too close in proximity to the cricopharyngeus muscle, hindering stent deployment. Failure to achieve technical success occurred in a further two patients when the stent was inserted distal to the stricture, requiring repositioning. Finally, stent insertion at the stricture site was not feasible in one patient who had previously undergone an oesophagectomy, despite the use of dilation beforehand.

**Table 4:** Technical and clinical success as reported in the included studies

Study Author	Technical success N (%)	Clinical success N (%)	Stent migration N (%)	Re-intervention N (%)
Canena et al. (2012) [13]	10 (100)	3 (30)	2 (20)	7 (70)
Dhar et al. (2014) [14]	9 (100)	0	–	9 (100)
Griffiths et al. (2012) [18]	6 (86)	3 (60)	0	2 (40)
Hirdes et al. (2012) [19]	26 (93)	9 (40)	3 (11)	–
Karakan et al. (2013) [20]	7 (100)	5 (100)	0	4 (80)
Kochhar et al. (2017) [21]	13 (100)	2 (15.4)	1 (7.6)	12 (92)
McCain et al. (2015) [22]	17 (94)	14 (77.8)	0	4 (22)
Nogales et al. (2017) [15]	12 (100)	8 (66.6)	0	4 (33.3)
Repici et al. (2010) [23]	21 (100)	9 (43)	2 (9.5)	11 (52)
Saeed et al. (2018) [24]	5 (100)	5 (100)	1 (20)	0
Sigounas et al. (2016) [25]	10 (100)	2 (20)	2 (20)	8 (80)
Van Boeckel et al. (2011) [16]	16 (85)	6 (33)	4 (22)	16 (42.1)
Van Hooft et al. (2011) [26]	10 (100)	6 (60)	0	4 (40)
Walter et al. (2018) [17]	32 (100)	15 (46.9)	1 (3.1)	4 (12.5)
Yano et al. (2017) [27]	18 (100)	12 (66.7)	0	–
Yano et al. (2022) [28]	29 (96.7)	4 (13.3)	0	4 (13.3)
Overall	239 (97.2)	103 (41.9)	16 (6.5)	89 (36.2)

Incidence of BDS stent migration occurred in 6.5% (95% CI = 3.4 – 9.6%) of participants, where the stent had migrated distally to the stricture site. Due to the biodegradable properties of the SX-ELLA BDS, stent migration does not require further endoscopic intervention to remove the stent. Despite this, surgery was necessary in one case to remove the stent, the reason for which was not disclosed. This case also resulted in the development of a post-surgical BOS. A case of one patient with a gastric bleed attributed to the migrated BDS into the stomach was also described. However, gastric migration of the BDS without any clinical consequences has also been reported.

When considering the safety profile of the SX-ELLA BDS, a total of 37 (15.0%, 95% CI = 10.5 – 19.5%) major clinical complications requiring intervention were reported, as demonstrated in Table 5. The most common of which were recurrent dysphagia following BDS insertion in 9 (3.7%) participants which required hospitalisation occurring 100 – 260 days post-BDS insertion. Severe thoracic pain occurred in 7 (2.8%) cases ‘immediately’ after stent insertion, rectified through opiate analgesics, such as pethidine. Food bolus obstruction was observed in 6 (2.3%) cases, requiring endoscopic cleaning of the BDS to clear the source of obstruction. Only one study stated the timeframe of this complication, reporting obstruction 74-day post-BDS insertion [26]. Major haemorrhage was noted in 5 (2.0%) participants and managed through blood product supplementation; the timing of this complication was not reported in the literature. Notably, 3 (1.2%) participants experienced tracheoesophageal fistula attributed to BDS insertion. Two of these cases occurred 95- and 96-days post-BDS insertion, both participants died following complications of the fistula, despite interventions, including tracheal repair, thoracotomy, tracheal stent insertion, and tracheostomy [17]. Other serious adverse events which were observed less often are detailed in Table 5.

**Table 5:** Major clinical complications as reported in the included studies

Complication	Cases, *N*
Recurrent dysphagia	9
Severe thoracic pain	7
Food bolus obstruction	6
Tracheoesophageal fistula	3
Oesophageal ulceration	1
Neurological event	1
Vascular event	1
Access site infection	1
Overall	37

## Discussion

This systematic review assesses the role of the SX-ELLA biodegradable stent for benign oesophageal strictures in adults. It builds on the five studies previously reviewed by Imaz-Iglesia et al. [29], who also stated the need for an updated review including the Walter et al. [17] RCT, which was not published at the time. Therefore, this systematic review includes studies published since the Imaz-Iglesia et al. review [29] and assesses a greater range of literature, through the inclusion of case series, which were not studied.

A total of 16 studies with 246 participants were analysed, where following the insertion of a BDS, 41.3% (95% CI = 35.7 – 48.1%) of participants experienced complete resolution of symptoms. The main symptom experienced was dysphagia, which decreased on average by 1.8 points (95% CI = 1.68 – 1.91) post-BDS insertion, assessed using the Ogilvie Dysphagia scale [9]. Current guidelines recommend the use of endoscopic dilation as the first-line management for BOS [4]. However, as repeated dilation procedures are required monthly with dysphagia recurrence, this becomes incredibly time-consuming for the patient, impacting their quality of life as well as having debatable cost-effectiveness for healthcare services. This review demonstrates that as re-intervention was only required in 36.2% (95% CI = 30.2–42.2%) of cases, BDS are a promising option for a one-step treatment for dysphagia caused by BOS.

Technical success was also high at 97.2% (95% CI = 95.1 – 99.3%), with an average procedure time of 30 min, making BDS a time-effective intervention. Stent migration occurred in 6.5% (95% CI = 3.4 – 9.6%) of participants, a rate considerably lower than both SEMS (30%) and SEPS (27%) [5, 6]. A key advantage of the BDS stent is that endoscopic removal is not required due to the natural hydrolysis of the stent. Furthermore, in the event of gastric migration, the low pH of the stomach increases the rate of BDS degradation [30]. Endoscopic dilation before stent insertion is an interesting factor when considering stent migration, with mixed results in the literature. Dilation is often used before stent insertion to allow widening of the stricture thus permitting stent insertion. However, Saeed et al. [24] reported increased BDS migration rates in participants who had undergone endoscopic dilation before BDS insertion. To study this effect, high-quality evidence such as RCTs that compare BDS insertion alone to endoscopic dilation with BDS insertion are required.

Alternative stent materials such as PC-SEMS have proved problematic in benign strictures, with tissue hyperplasia being a notable complication, decreasing clinical effectiveness and complicating stent removal [5]. In this review, BDS stents led to major complications in 37 (15.0%, 95% CI = 10.5 – 19.5%) incidences. Whilst most of these complications were managed through timely intervention, two cases of tracheoesophageal fistula attributed to BDS insertion occurred in Walter et al. 95- and 96-day post-BDS insertion, with both cases leading to death [17]. Although this complication was rare, it is important to analyse any factors present in these participants which increased the likelihood of fistula formation and thus whether future patients at risk of such complications can be identified before the procedure.

Regarding the complication of severe thoracic pain, this was reported to a greater extent in the studies which used the largest stent diameter of 25 mm. Therefore, it is possible that the higher radial force the larger stents exerted contributed to the increased rates of post-insertion thoracic pain [19]. However, this is only a single factor and others contributing to the increased risk of thoracic pain must also be identified. Despite this, the safety profile of BDS insertion is still superior to that of oesophageal surgery. The various surgical complications can hinder effective and safe intervention in older patients and those with co-morbidities, such as ischaemic heart disease and type 2 diabetes mellitus, both of which are increasing in prevalence.

The limitations of this systematic review must also be addressed. Despite a thorough literature search and use of studies published as recent as 2022, there are a lack of high-quality evidence, with only one RCT and one pilot RCT having been conducted. To create robust conclusions regarding the effectiveness of BDS to treat BOS, RCTs must be conducted where BDS insertion is compared to other methods of intervention, such as endoscopic dilation, SEMS, SEPS, and potentially oesophageal surgery. Furthermore, a comparison to FC-SEMS with the additional elements available such as the StentFix OTSC device or endoscopic suture fixation would be highly informative. Such features were developed to reduce complications of stent migration in SEMS. Therefore, a comparison with these additions would allow for a more clinically valuable insight into effectiveness.

Additionally, the studies’ small sample sizes are evident, with under-recruitment being cited as the reason for Dhar et al.’s RCT being abandoned [14]. Consequently, the results drawn from these small samples can be highly variable and prone to bias. Publication bias may also affect the studies, with researchers reporting more favourable results, for example, representing averages through medians that appear unaffected by skewed data. A complete meta-analysis was prevented by the high heterogeneity of the studies, caused by differences in clinical factors, lack of randomisation, and early termination of studies.

A further limitation to this review is that sub-group analysis according to stricture pathogenesis was not possible. It would be valuable to assess which types of benign strictures benefitted greatest from the use of BDS and if time spent dysphagia free differed between these sub-groups. This analysis was unfortunately not possible as despite the studies reporting the underlying stricture pathogenesis in participants, when reporting the results, this was done in tandem, with all strictures types reported together. Therefore, it is not possible to know which strictures had better or worse rates of clinical and technical effectiveness or complications. It would be increasingly informative if future studies could highlight results according to stricture pathogenesis so such an assessment can be undertaken.

Furthermore, this review focuses on the use of BDS to treat benign oesophageal strictures in adults, meaning the findings are only applicable to those > 18 years. The primary outcome of dysphagia was measured using the Ogilvie Dysphagia scale, which despite being a self-report tool, is superior to alternatives, such as the Watson and Goldschmid scales [31]. However, the Ogilvie Dysphagia scale was originally developed to assess malignant dysphagia, as reflected in Persson et al.’s validation study [31]. Therefore, despite the researchers in ten of the included studies deeming the tool to be valid, the use of the scale for benign dysphagia is yet to be validated. Finally, the follow-up period in the studies varied greatly, with the longest being 33.3 months [15] meaning greater evidence is required to fully understand the long-term effectiveness of the SX-ELLA BDS to treat benign oesophageal strictures.

## Conclusion

To conclude, this systematic review shows the high technical and moderate clinical success rate of the SX-ELLA biodegradable stent to treat benign oesophageal strictures in adults.

## Registration and protocol

This systematic review was prospectively registered on PROSPERO (registration number: CRD42022307985 [32] and reported according to the Preferred Reporting Items for Systematic Reviews and Meta-Analysis (PRISMA) checklist.

## Supplementary Information

Below is the link to the electronic supplementary material.

Supplementary file1 (DOCX 49 KB)

Supplementary file2 (DOCX 50 KB)

Supplementary file3 (DOCX 49 KB)

Supplementary file4 (DOCX 16 KB)

Supplementary file5 (DOCX 16 KB)

Supplementary file6 (DOCX 16 KB)

Supplementary file7 (DOCX 16 KB)

Supplementary file8 (DOCX 16 KB)

Supplementary file9 (DOCX 16 KB)

Supplementary file10 (DOCX 16 KB)

Supplementary file11 (DOCX 16 KB)

Supplementary file12 (DOCX 16 KB)

Supplementary file13 (DOCX 16 KB)

Supplementary file14 (DOCX 16 KB)

Supplementary file15 (DOCX 41 KB)

Supplementary file16 (DOCX 41 KB)

Supplementary file17 (JPG 70 KB)
