# Supplementary material for: SX-ELLA biodegradable stent for benign oesophageal strictures: a systematic review and proportion meta-analysis
Source: Surg Endosc. 2022 Dec 8;37(4):2476–84. doi: 10.1007/s00464-022-09767-w (PMC10082093; doi:10.1007/s00464-022-09767-w)
Supplement: Supplementary file 2 — Supplementary file2 (DOCX 50 KB) [file 464_2022_9767_MOESM2_ESM.docx]

# ROBINS-I tool (Stage II): For each study

Nogales Ó, Clemente A, Caballero-Marcos A, García-Lledó J, Pérez-Carazo L, Merino B et al. Endoscopically placed stents: a useful alternative for the management of refractory benign cervical esophageal stenosis. Revista Española de Enfermedades Digestivas. 2017;109.

## Specify a target randomized trial specific to the study

| Design | Individually randomized / Cluster randomized / Matched (e.g. cross-over) |
| --- | --- |
| Participants | 12 participants with refractory, benign oesophageal strictures |
| Experimental intervention | BDS |
| Comparator | FCSEMS |

## Is your aim for this study…?

| X | to assess the effect of *assignment to* intervention |
| --- | --- |
| □ | to assess the effect of *starting and adhering to* intervention |

## Specify the outcome

Specify which outcome is being assessed for risk of bias (typically from among those earmarked for the Summary of Findings table). Specify whether this is a proposed benefit or harm of intervention.

| - Clinical effectiveness of stent insertion, assessing dysphagia free status post-intervention. |
| --- |

## Specify the numerical result being assessed

In case of multiple alternative analyses being presented, specify the numeric result (e.g. RR = 1.52 (95% CI 0.83 to 2.77) and/or a reference (e.g. to a table, figure or paragraph) that uniquely defines the result being assessed.

| - Refer to the results regarding time spent dysphagia free. |
| --- |

## Risk of bias assessment

Responses underlined in green are potential markers for low risk of bias, and responses in red are potential markers for a risk of bias. Where questions relate only to sign posts to other questions, no formatting is used.

|  | **Signalling questions** | **Description** | **Response options** |
| --- | --- | --- | --- |
| **Bias due to confounding** | | | |
|  | 1.1 Is there potential for confounding of the effect of intervention in this study?  **If N/PN to 1.1:** the study can be considered to be at low risk of bias due to confounding and no further signalling questions need be considered | - Effect of balloon dilation prior to stent insertion, not all participants had dilation, which could have affected the clinical outcome of results, e.g. participants receiving dilation were had a greater feeling of being dysphagia free. - Each intervention was completed on a case by case basis, therefore a homogenous approach was not used for all participants during stent insertion such as whether dilation was performed prior to the insertion and whether the endoscopy was done under fluoroscopic guidance. | Y / PY / PN / N |
|  | **If Y/PY to 1.1**: determine whether there is a need to assess time-varying confounding: |  |  |
|  | 1.2. Was the analysis based on splitting participants’ follow up time according to intervention received?  **If N/PN**, answer questions relating to baseline confounding (1.4 to 1.6)  **If Y/PY**, go to question 1.3. | - No, the participants’ follow-up time was not dependent on which intervention was received. | NA / Y / PY / PN / N / NI |
|  | 1.3. Were intervention discontinuations or switches likely to be related to factors that are prognostic for the outcome?  **If N/PN**, answer questions relating to baseline confounding (1.4 to 1.6)  **If Y/PY**, answer questions relating to both baseline and time-varying confounding (1.7 and 1.8) |  | NA / Y / PY / PN / N / NI |

|  | **Questions relating to baseline confounding only** | | |
| --- | --- | --- | --- |
|  | 1.4. Did the authors use an appropriate analysis method that controlled for all the important confounding domains? | - Reference is made to statistical analysis performed, however it does not state whether this was able to control for confounding domains. | NA / Y / PY / PN / N / NI |
|  | 1.5. **If Y/PY to 1.4**: Were confounding domains that were controlled for measured validly and reliably by the variables available in this study? |  | NA / Y / PY / PN / N / NI |
|  | 1.6. Did the authors control for any post-intervention variables that could have been affected by the intervention? | - There was no control of post-intervention variables following the stent insertion. | NA / Y / PY / PN / N / NI |
|  | **Questions relating to baseline and time-varying confounding** | |  |
|  | 1.7. Did the authors use an appropriate analysis method that controlled for all the important confounding domains and for time-varying confounding? | - There is no reference made as to whether the confounding domains were controlled for both baseline and time-varying domains, therefore this is unclear. | NA / Y / PY / PN / N / NI |
|  | 1.8. **If Y/PY to 1.7**: Were confounding domains that were controlled for measured validly and reliably by the variables available in this study? |  | NA / Y / PY / PN / N / NI |
|  | **Risk of bias judgement** |  | Low / Moderate / Serious / Critical / NI |
|  | Optional: What is the predicted direction of bias due to confounding? |  | Favours experimental / Favours comparator / Unpredictable |

| **Bias in selection of participants into the study** | | | |
| --- | --- | --- | --- |
|  | 2.1. Was selection of participants into the study (or into the analysis) based on participant characteristics observed after the start of intervention?  **If N/PN to 2.1:** go to 2.4 | - Despite being a retrospective analysis, the study included all patients who had received stent insertion over the course of seven years, therefore the selection of participants was not done dependent on their outcome. | Y / PY / PN / N / NI |
|  | 2.2. **If Y/PY to 2.1**: Were the post-intervention variables that influenced selection likely to be associated with intervention?  2.3 **If Y/PY to 2.2**: Were the post-intervention variables that influenced selection likely to be influenced by the outcome or a cause of the outcome? |  | NA / Y / PY / PN / N / NI  NA / Y / PY / PN / N / NI |
|  | 2.4. Do start of follow-up and start of intervention coincide for most participants? | - The retrospective analysis means that the time period in which participants received their intervention and the beginning of follow-up differed, as they received the intervention potentially years apart. However, the time between stent insertion and follow-up was the same between all participants, all were followed-up immediately after surgery, and then at eight weeks where removal of the metal stent occurred and degradation of the biodegradable stent. | Y / PY / PN / N / NI |
|  | 2.5. **If Y/PY to 2.2 and 2.3, or N/PN to 2.4**: Were adjustment techniques used that are likely to correct for the presence of selection biases? |  | NA / Y / PY / PN / N / NI |
|  | **Risk of bias judgement** |  | Low / Moderate / Serious / Critical / NI |
|  | Optional: What is the predicted direction of bias due to selection of participants into the study? |  | Favours experimental / Favours comparator / Towards null /Away from null / Unpredictable |

| **Bias in classification of interventions** | | | |
| --- | --- | --- | --- |
|  | 3.1 Were intervention groups clearly defined? | - Yes, participants either received the FC-SEMS or the BDS | Y / PY / PN / N / NI |
|  | 3.2 Was the information used to define intervention groups recorded at the start of the intervention? | - Yes, the different interventions were pre-defined before analysis. However, this was not before the start of intervention as the study was performed using retrospective analysis, so the interventions had already been performed before the intervention groups were defined. | Y / PY / PN / N / NI |
|  | 3.3 Could classification of intervention status have been affected by knowledge of the outcome or risk of the outcome? | - No, the interventions were very clear and defined, participants either received a FC-SEMS or a BDS, therefore classification was not changed by knowledge of the outcome. | Y / PY / PN / N / NI |
|  | **Risk of bias judgement** |  | Low / Moderate / Serious / Critical / NI |
|  | Optional: What is the predicted direction of bias due to classification of interventions? |  | Favours experimental / Favours comparator / Towards null /Away from null / Unpredictable |

| **Bias due to deviations from intended interventions** | | | |
| --- | --- | --- | --- |
|  | **If your aim for this study is to assess the effect of assignment to intervention, answer questions 4.1 and 4.2** | |  |
|  | 4.1. Were there deviations from the intended intervention beyond what would be expected in usual practice? | - Partly yes as there was no homogenous procedure performed, stents were inserted based on availability rather than patient suitability. Pre-insertion dilation was also performed on only select patients. | Y / PY / PN / N / NI |
|  | 4.2. **If Y/PY to 4.1**: Were these deviations from intended intervention unbalanced between groups *and* likely to have affected the outcome? | - The participants who received pre-insertion endoscopic dilation could have had their outcome affected but a definite causation effect between dilation and clinical effectiveness is not known. | NA / Y / PY / PN / N / NI |
|  | **If your aim for this study is to assess the effect of starting and adhering to intervention, answer questions 4.3 to 4.6** | |  |
|  | 4.3. Were important co-interventions balanced across intervention groups? | - No, the co-intervention of pre-insertion endoscopic dilation was not balanced between the two intervention groups. | Y / PY / PN / N / NI |
|  | 4.4. Was the intervention implemented successfully for most participants? | - One participant underwent an initially unsuccessful stent insertion, which had to be resolved through removal of the stent and subsequent replacement. | Y / PY / PN / N / NI |
|  | 4.5. Did study participants adhere to the assigned intervention regimen? |  | Y / PY / PN / N / NI |
|  | 4.6. **If N/PN to 4.3, 4.4 or 4.5**: Was an appropriate analysis used to estimate the effect of starting and adhering to the intervention? |  | NA / Y / PY / PN / N / NI |
|  | **Risk of bias judgement** |  | Low / Moderate / Serious / Critical / NI |
|  | Optional: What is the predicted direction of bias due to deviations from the intended interventions? |  | Favours experimental / Favours comparator / Towards null /Away from null / Unpredictable |

| **Bias due to missing data** | | | |
| --- | --- | --- | --- |
|  | 5.1 Were outcome data available for all, or nearly all, participants? |  | Y / PY / PN / N / NI |
|  | 5.2 Were participants excluded due to missing data on intervention status? | - None of the participants who received a stent insertion were excluded from the study. | Y / PY / PN / N / NI |
|  | 5.3 Were participants excluded due to missing data on other variables needed for the analysis? | - None of the participants who received a stent insertion were excluded from the study. | Y / PY / PN / N / NI |
|  | 5.4 **If PN/N to 5.1, or Y/PY to 5.2 or 5.3**: Are the proportion of participants and reasons for missing data similar across interventions? |  | NA / Y / PY / PN / N / NI |
|  | 5.5 **If PN/N to 5.1, or Y/PY to 5.2 or 5.3**: Is there evidence that results were robust to the presence of missing data? |  | NA / Y / PY / PN / N / NI |
|  | **Risk of bias judgement** |  | Low / Moderate / Serious / Critical / NI |
|  | Optional: What is the predicted direction of bias due to missing data? |  | Favours experimental / Favours comparator / Towards null /Away from null / Unpredictable |

| **Bias in measurement of outcomes** | | | |
| --- | --- | --- | --- |
|  | 6.1 Could the outcome measure have been influenced by knowledge of the intervention received? | - No, as the outcomes had already occurred as this study used a retrospective analysis, therefore the clinicians conducting this study were not able to influence the outcomes. | Y / PY / PN / N / NI |
|  | 6.2 Were outcome assessors aware of the intervention received by study participants? | - Yes, assessors were aware of the interventions received as the retrospective analysis meant looking at participants over the seven years who had had either a FC-SEMS or BDS inserted. | Y / PY / PN / N / NI |
|  | 6.3 Were the methods of outcome assessment comparable across intervention groups? | - Yes, outcome measures were standardised between groups | Y / PY / PN / N / NI |
|  | 6.4 Were any systematic errors in measurement of the outcome related to intervention received? |  | Y / PY / PN / N / NI |
|  | **Risk of bias judgement** |  | Low / Moderate / Serious / Critical / NI |
|  | Optional: What is the predicted direction of bias due to measurement of outcomes? |  | Favours experimental / Favours comparator / Towards null /Away from null / Unpredictable |

| **Bias in selection of the reported result** | | | |
| --- | --- | --- | --- |
|  | Is the reported effect estimate likely to be selected, on the basis of the results, from... | - The outcome domains were only measured using single assessments per outcome. Statistical tests were also performed to determine the probability of the outcomes being due to chance or not. |  |
|  | 7.1. ... multiple outcome *measurements* within the outcome domain? |  | Y / PY / PN / N / NI |
|  | 7.2 ... multiple *analyses* of the intervention-outcome relationship? | - The intervention-outcome analysis was not subject to multiple analyses. | Y / PY / PN / N / NI |
|  | 7.3 ... different *subgroups*? | - There was no sub-group analysis performed. | Y / PY / PN / N / NI |
|  | **Risk of bias judgement** |  | Low / Moderate / Serious / Critical / NI |
|  | Optional: What is the predicted direction of bias due to selection of the reported result? |  | Favours experimental / Favours comparator / Towards null /Away from null / Unpredictable |

| **Overall bias** | | | |
| --- | --- | --- | --- |
|  | **Risk of bias judgement** | Due to the serious bias rating regarding the bias domain of intended interventions, overall bias is judged as being serious. | Low / Moderate / Serious / Critical / NI |
|  | Optional: What is the overall predicted direction of bias for this outcome? |  | Favours experimental / Favours comparator / Towards null /Away from null / Unpredictable |


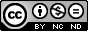


This work is licensed under a [Creative Commons Attribution-NonCommercial-NoDerivatives 4.0 International License](http://creativecommons.org/licenses/by-nc-nd/4.0/).
