# Supplementary material for: SX-ELLA biodegradable stent for benign oesophageal strictures: a systematic review and proportion meta-analysis
Source: Surg Endosc. 2022 Dec 8;37(4):2476–84. doi: 10.1007/s00464-022-09767-w (PMC10082093; doi:10.1007/s00464-022-09767-w)
Supplement: Supplementary file 6 — Supplementary file6 (DOCX 16 KB) [file 464_2022_9767_MOESM6_ESM.docx]

**JBI Critical Appraisal Checklist for Case Series**

Reviewer FR______________ Date____03/01/2021___________________________

Author___KARAKAN ET AL_________________ Year___2013______ Record Number_________

|  | Yes | No | Unclear | Not applicable |
| --- | --- | --- | --- | --- |
| - Were there clear criteria for inclusion in the case series? | □ | X | □ | □ |
| - Was the condition measured in a standard, reliable way for all participants included in the case series? | X | □ | □ | □ |
| - Were valid methods used for identification of the condition for all participants included in the case series? | X | □ | □ | □ |
| - Did the case series have consecutive inclusion of participants? | X | □ | □ | □ |
| - Did the case series have complete inclusion of participants? | X | □ | □ | □ |
| - Was there clear reporting of the demographics of the participants in the study? | □ | □ | X | □ |
| - Was there clear reporting of clinical information of the participants? | □ | X | □ | □ |
| - Were the outcomes or follow up results of cases clearly reported? | X | □ | □ | □ |
| - Was there clear reporting of the presenting site(s)/clinic(s) demographic information? | □ | □ | X | □ |
| - Was statistical analysis appropriate? | □ | □ | X | □ |

Overall appraisal: Include X Exclude □ Seek further info □

Comments (Including reason for exclusion)

___THIS CASE SERIES HAS A PROBABLE RISK OF BIAS, IT IS STILL TO BE INCLUDED IN THE STUDIES WHILST TAKING THIS RISK OF BIAS IN TO ACCOUNT _____________________________________________________________________________________________________________________________________________________________________________________________
