# Supplementary material for: SX-ELLA biodegradable stent for benign oesophageal strictures: a systematic review and proportion meta-analysis
Source: Surg Endosc. 2022 Dec 8;37(4):2476–84. doi: 10.1007/s00464-022-09767-w (PMC10082093; doi:10.1007/s00464-022-09767-w)
Supplement: Supplementary file 15 — Supplementary file15 (DOCX 41 KB) [file 464_2022_9767_MOESM15_ESM.docx]

Revised Cochrane risk-of-bias tool for randomized trials (RoB 2)

TEMPLATE FOR COMPLETION

Edited by Julian PT Higgins, Jelena Savović, Matthew J Page, Jonathan AC Sterne
on behalf of the RoB2 Development Group

**Version of 22 August 2019**

The development of the RoB 2 tool was supported by the MRC Network of Hubs for Trials Methodology Research (MR/L004933/2- N61), with the support of the host MRC ConDuCT-II Hub (Collaboration and innovation for Difficult and Complex randomised controlled Trials In Invasive procedures - MR/K025643/1), by MRC research grant MR/M025209/1, and by a grant from The Cochrane Collaboration.


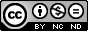


This work is licensed under a [Creative Commons Attribution-NonCommercial-NoDerivatives 4.0 International License](http://creativecommons.org/licenses/by-nc-nd/4.0/).

| **Study details**   \| **Reference** \| Dhar A. Biodegradable stent or balloon dilatation for benign oesophageal stricture: Pilot randomised controlled trial. World Journal of Gastroenterology. 2014;20(48):18199. \| \| --- \| --- \|   **Study design**   \| X \| Individually-randomized parallel-group trial \| \| --- \| --- \| \| □ \| Cluster-randomized parallel-group trial \| \| □ \| Individually randomized cross-over (or other matched) trial \|   **For the purposes of this assessment, the interventions being compared are defined as**   \| Experimental: \| BDS Insertion \| Comparator: \| Balloon dilation \| \| --- \| --- \| --- \| --- \|  \| **Specify which outcome is being assessed for risk of bias** \| Primary end point: dysphagia score \| \| --- \| --- \|  \| **Specify the numerical result being assessed.** In case of multiple alternative analyses being presented, specify the numeric result (e.g. RR = 1.52 (95% CI 0.83 to 2.77) and/or a reference (e.g. to a table, figure or paragraph) that uniquely defines the result being assessed. \| See results paragraph of study regarding post-intervention dysphagia scores. \| \| --- \| --- \|   **Is the review team’s aim for this result…?**   \| X \| to assess the effect of *assignment to intervention* (the ‘intention-to-treat’ effect) \| \| --- \| --- \| \| □ \| to assess the effect of *adhering to intervention* (the ‘per-protocol’ effect) \|   **If the aim is to assess the effect of *adhering to intervention***, select the deviations from intended intervention that should be addressed (at least one must be checked):  □ occurrence of non-protocol interventions  □ failures in implementing the intervention that could have affected the outcome  □ non-adherence to their assigned intervention by trial participants  **Which of the following sources were obtained to help inform the risk-of-bias assessment? (tick as many as apply)**  X Journal article(s) with results of the trial  □ Trial protocol  □ Statistical analysis plan (SAP)  □ Non-commercial trial registry record (e.g. ClinicalTrials.gov record)  □ Company-owned trial registry record (e.g. GSK Clinical Study Register record)  □ “Grey literature” (e.g. unpublished thesis)  □ Conference abstract(s) about the trial  □ Regulatory document (e.g. Clinical Study Report, Drug Approval Package)  □ Research ethics application  □ Grant database summary (e.g. NIH RePORTER or Research Councils UK Gateway to Research)  □ Personal communication with trialist  □ Personal communication with the sponsor |
| --- | --- | --- | --- | --- | --- | --- | --- | --- | --- | --- | --- | --- | --- | --- | --- | --- | --- | --- | --- | --- |

## Risk of bias assessment

Responses underlined in green are potential markers for low risk of bias, and responses in red are potential markers for a risk of bias. Where questions relate only to sign posts to other questions, no formatting is used.

**Domain 1: Risk of bias arising from the randomization process**

| **Signalling questions** | **Comments** | **Response options** |
| --- | --- | --- |
| **1.1 Was the allocation sequence random?** | Randomisation was performed in blocks of four in a 1:1 ratio, using a web-based system.  Yes, the study specifies the clinician responsible for participant recruitment provided patient details before allocation of interventions were disclosed, therefore allowing concealment. | Y / PY / PN / N / NI |
| **1.2 Was the allocation sequence concealed until participants were enrolled and assigned to interventions?** |  | Y / PY / PN / N / NI |
| **1.3 Did baseline differences between intervention groups suggest a problem with the randomization process?** | Participants were similar in demographics, disease features and co-morbidities following randomisation. | Y / PY / PN / N / NI |
| **Risk-of-bias judgement** |  | Low / High / Some concerns |
| Optional: What is the predicted direction of bias arising from the randomization process? |  | NA / Favours experimental / Favours comparator / Towards null /Away from null / Unpredictable |

Domain 2: Risk of bias due to deviations from the intended interventions (*effect of assignment to intervention*)

| **Signalling questions** | **Comments** | **Response options** |
| --- | --- | --- |
| **2.1. Were participants aware of their assigned intervention during the trial?** | Yes, participants were aware of which endoscopic procedure they would be receiving due to the need for informed consent prior to the procedure.  Yes, the healthcare professionals delivering the endoscopic procedures were aware of the assigned intervention, it is not possible to blind this due to the nature of the interventions. However, the clinician who interpretated the outcomes of each arm was blinded to the intervention the participant received. | Y / PY / PN / N / NI |
| **2.2. Were carers and people delivering the interventions aware of participants' assigned intervention during the trial?** |  | Y / PY / PN / N / NI |
| **2.3. If Y/PY/NI to 2.1 or 2.2: Were there deviations from the intended intervention that arose because of the trial context?** |  | NA / Y / PY / PN / N / NI |
| **2.4 If Y/PY to 2.3: Were these deviations likely to have affected the outcome?** |  | NA / Y / PY / PN / N / NI |
| **2.5. If Y/PY/NI to 2.4: Were these deviations from intended intervention balanced between groups?** |  | NA / Y / PY / PN / N / NI |
| **2.6 Was an appropriate analysis used to estimate the effect of assignment to intervention?** | Yes, the intention to treat analysis principles were followed throughout. | Y / PY / PN / N / NI |
| **2.7 If N/PN/NI to 2.6: Was there potential for a substantial impact (on the result) of the failure to analyse participants in the group to which they were randomized?** |  | NA / Y / PY / PN / N / NI |
| **Risk-of-bias judgement** |  | Low / High / Some concerns |
| Optional: What is the predicted direction of bias due to deviations from intended interventions? |  | NA / Favours experimental / Favours comparator / Towards null /Away from null / Unpredictable |

Domain 2: Risk of bias due to deviations from the intended interventions (*effect of adhering to intervention*)

| **Signalling questions** | **Comments** | **Response options** |
| --- | --- | --- |
| **2.1. Were participants aware of their assigned intervention during the trial?** |  | Y / PY / PN / N / NI |
| **2.2. Were carers and people delivering the interventions aware of participants' assigned intervention during the trial?** |  | Y / PY / PN / N / NI |
| **2.3. [If applicable:] If Y/PY/NI to 2.1 or 2.2: Were important non-protocol interventions balanced across intervention groups?** |  | NA / Y / PY / PN / N / NI |
| **2.4. [If applicable:] Were there failures in implementing the intervention that could have affected the outcome?** |  | NA / Y / PY / PN / N / NI |
| **2.5. [If applicable:] Was there non-adherence to the assigned intervention regimen that could have affected participants’ outcomes?** |  | NA / Y / PY / PN / N / NI |
| **2.6. If N/PN/NI to 2.3, or Y/PY/NI to 2.4 or 2.5: Was an appropriate analysis used to estimate the effect of adhering to the intervention?** |  | NA / Y / PY / PN / N / NI |
| **Risk-of-bias judgement** |  | Low / High / Some concerns |
| Optional: What is the predicted direction of bias due to deviations from intended interventions? |  | NA / Favours experimental / Favours comparator / Towards null /Away from null / Unpredictable |

Domain 3: Missing outcome data

| **Signalling questions** | **Comments** | **Response options** |
| --- | --- | --- |
| **3.1 Were data for this outcome available for all, or nearly all, participants randomized?** | Yes, nearly all participants had their data analysed, those who did not where lost to follow-up or did not receive the intervention as disclosed in the RCT consort flow diagram. | Y / PY / PN / N / NI |
| **3.2 If N/PN/NI to 3.1: Is there evidence that the result was not biased by missing outcome data?** |  | NA / Y / PY / PN / N |
| **3.3 If N/PN to 3.2: Could missingness in the outcome depend on its true value?** |  | NA / Y / PY / PN / N / NI |
| **3.4 If Y/PY/NI to 3.3: Is it likely that missingness in the outcome depended on its true value?** |  | NA / Y / PY / PN / N / NI |
| **Risk-of-bias judgement** |  | Low / High / Some concerns |
| Optional: What is the predicted direction of bias due to missing outcome data? |  | NA / Favours experimental / Favours comparator / Towards null /Away from null / Unpredictable |

Domain 4: Risk of bias in measurement of the outcome

| **Signalling questions** | **Comments** | **Response options** |
| --- | --- | --- |
| **4.1 Was the method of measuring the outcome inappropriate?** | No, dysphagia scores were used to manage primary outcomes of symptom resolution, statistical differences regarding this score were also calculated across a range of sensitivities. | Y / PY / PN / N / NI |
| **4.2 Could measurement or ascertainment of the outcome have differed between intervention groups?** | No, any differences in baseline characteristics and site of treatment were accounted for in the calculations for significance and adjusted to account for this.. | Y / PY / PN / N / NI |
| **4.3 If N/PN/NI to 4.1 and 4.2: Were outcome assessors aware of the intervention received by study participants?** |  | NA / Y / PY / PN / N / NI |
| **4.4 If Y/PY/NI to 4.3: Could assessment of the outcome have been influenced by knowledge of intervention received?** |  | NA / Y / PY / PN / N / NI |
| **4.5 If Y/PY/NI to 4.4:** **Is it likely that assessment of the outcome was influenced by knowledge of intervention received?** |  | NA / Y / PY / PN / N / NI |
| **Risk-of-bias judgement** |  | Low / High / Some concerns |
| Optional: What is the predicted direction of bias in measurement of the outcome? |  | NA / Favours experimental / Favours comparator / Towards null /Away from null / Unpredictable |

Domain 5: Risk of bias in selection of the reported result

| **Signalling questions** | **Comments** | **Response options** |
| --- | --- | --- |
| **5.1 Were the data that produced this result analysed in accordance with a pre-specified analysis plan that was finalized before unblinded outcome data were available for analysis?** | Yes, the intention to treat analysis and statistical methods to be used for the trial were determined before the outcome data was available for analysis. | Y / PY / PN / N / NI |
| **Is the numerical result being assessed likely to have been selected, on the basis of the results, from...** |  |  |
| **5.2. ... multiple eligible outcome measurements (e.g. scales, definitions, time points) within the outcome domain?** | The same scales and definitions for the outcome measurements were maintained throughout the RCT. | Y / PY / PN / N / NI |
| **5.3 ... multiple eligible analyses of the data?** | Analyses of the same data would produce the same results when using the analysis techniques that were detailed in the RCT. The researchers also adjusted sensitivity calculations for baseline characteristics to ensure results were still eligible. | Y / PY / PN / N / NI |
| **Risk-of-bias judgement** |  | Low / High / Some concerns |
| Optional: What is the predicted direction of bias due to selection of the reported result? |  | NA / Favours experimental / Favours comparator / Towards null /Away from null / Unpredictable |

Overall risk of bias

| **Risk-of-bias judgement** | Overall bias must be ranked as ‘some concerns’ due to the bias concerns detailed in Domain 2. | Low / High / Some concerns |
| --- | --- | --- |
| Optional: What is the overall predicted direction of bias for this outcome? |  | NA / Favours experimental / Favours comparator / Towards null /Away from null / Unpredictable |


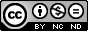


This work is licensed under a [Creative Commons Attribution-NonCommercial-NoDerivatives 4.0 International License](http://creativecommons.org/licenses/by-nc-nd/4.0/).
