# Supplementary material for: SX-ELLA biodegradable stent for benign oesophageal strictures: a systematic review and proportion meta-analysis
Source: Surg Endosc. 2022 Dec 8;37(4):2476–84. doi: 10.1007/s00464-022-09767-w (PMC10082093; doi:10.1007/s00464-022-09767-w)
Supplement: Supplementary file 16 — Supplementary file16 (DOCX 41 KB) [file 464_2022_9767_MOESM16_ESM.docx]

Revised Cochrane risk-of-bias tool for randomized trials (RoB 2)

TEMPLATE FOR COMPLETION

Edited by Julian PT Higgins, Jelena Savović, Matthew J Page, Jonathan AC Sterne
on behalf of the RoB2 Development Group

**Version of 22 August 2019**

The development of the RoB 2 tool was supported by the MRC Network of Hubs for Trials Methodology Research (MR/L004933/2- N61), with the support of the host MRC ConDuCT-II Hub (Collaboration and innovation for Difficult and Complex randomised controlled Trials In Invasive procedures - MR/K025643/1), by MRC research grant MR/M025209/1, and by a grant from The Cochrane Collaboration.


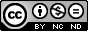


This work is licensed under a [Creative Commons Attribution-NonCommercial-NoDerivatives 4.0 International License](http://creativecommons.org/licenses/by-nc-nd/4.0/).

| **Study details**   \| **Reference** \| Walter D, van den Berg M, Hirdes M, Vleggaar F, Repici A, Deprez P et al. Dilation or biodegradable stent placement for recurrent benign esophageal strictures: a randomized controlled trial. Endoscopy. 2018;50(12):1146-1155. \| \| --- \| --- \|   **Study design**   \| X \| Individually-randomized parallel-group trial \| \| --- \| --- \| \| □ \| Cluster-randomized parallel-group trial \| \| □ \| Individually randomized cross-over (or other matched) trial \|   **For the purposes of this assessment, the interventions being compared are defined as**   \| Experimental: \| SX-ELLA biodegradable stent \| Comparator: \| Endoscopic dilation \| \| --- \| --- \| --- \| --- \|  \| **Specify which outcome is being assessed for risk of bias** \| Primary end-point of dilation for stricture recurrence \| \| --- \| --- \|  \| **Specify the numerical result being assessed.** In case of multiple alternative analyses being presented, specify the numeric result (e.g. RR = 1.52 (95% CI 0.83 to 2.77) and/or a reference (e.g. to a table, figure or paragraph) that uniquely defines the result being assessed. \| Paragraphs assessing the primary end-point of dilation for stricture recurrence \| \| --- \| --- \|   **Is the review team’s aim for this result…?**   \| X \| to assess the effect of *assignment to intervention* (the ‘intention-to-treat’ effect) \| \| --- \| --- \| \| □ \| to assess the effect of *adhering to intervention* (the ‘per-protocol’ effect) \|   **If the aim is to assess the effect of *adhering to intervention***, select the deviations from intended intervention that should be addressed (at least one must be checked):  □ occurrence of non-protocol interventions  □ failures in implementing the intervention that could have affected the outcome  □ non-adherence to their assigned intervention by trial participants  **Which of the following sources were obtained to help inform the risk-of-bias assessment? (tick as many as apply)**  X Journal article(s) with results of the trial  X Trial protocol  □ Statistical analysis plan (SAP)  □ Non-commercial trial registry record (e.g. ClinicalTrials.gov record)  □ Company-owned trial registry record (e.g. GSK Clinical Study Register record)  □ “Grey literature” (e.g. unpublished thesis)  □ Conference abstract(s) about the trial  □ Regulatory document (e.g. Clinical Study Report, Drug Approval Package)  □ Research ethics application  □ Grant database summary (e.g. NIH RePORTER or Research Councils UK Gateway to Research)  □ Personal communication with trialist  □ Personal communication with the sponsor |
| --- | --- | --- | --- | --- | --- | --- | --- | --- | --- | --- | --- | --- | --- | --- | --- | --- | --- | --- | --- | --- |

## Risk of bias assessment

Responses underlined in green are potential markers for low risk of bias, and responses in red are potential markers for a risk of bias. Where questions relate only to sign posts to other questions, no formatting is used.

**Domain 1: Risk of bias arising from the randomization process**

| **Signalling questions** | **Comments** | **Response options** |
| --- | --- | --- |
| **1.1 Was the allocation sequence random?** | Block randomisation was performed using a centralised computerised system, in a ratio of 1:1.  Allocation sequence concealment was upheld, as a centralised system away from the site of the RCT was used to conduct the assignment of participants. | Y / PY / PN / N / NI |
| **1.2 Was the allocation sequence concealed until participants were enrolled and assigned to interventions?** |  | Y / PY / PN / N / NI |
| **1.3 Did baseline differences between intervention groups suggest a problem with the randomization process?** | No, baseline differences of patient characteristics and stricture features were appropriately similar. | Y / PY / PN / N / NI |
| **Risk-of-bias judgement** |  | Low / High / Some concerns |
| Optional: What is the predicted direction of bias arising from the randomization process? |  | NA / Favours experimental / Favours comparator / Towards null /Away from null / Unpredictable |

Domain 2: Risk of bias due to deviations from the intended interventions (*effect of assignment to intervention*)

| **Signalling questions** | **Comments** | **Response options** |
| --- | --- | --- |
| **2.1. Were participants aware of their assigned intervention during the trial?** | Yes, participants were aware of which endoscopic procedure they would be receiving due to the need for informed consent prior to the procedure.  Yes, the healthcare professionals delivering the endoscopic procedures were aware of the assigned intervention, it is not possible to blind this due to the nature of the interventions. | Y / PY / PN / N / NI |
| **2.2. Were carers and people delivering the interventions aware of participants' assigned intervention during the trial?** |  | Y / PY / PN / N / NI |
| **2.3. If Y/PY/NI to 2.1 or 2.2: Were there deviations from the intended intervention that arose because of the trial context?** | No deviations from the intended intervention occurred and all patients received their assigned intervention. | NA / Y / PY / PN / N / NI |
| **2.4 If Y/PY to 2.3: Were these deviations likely to have affected the outcome?** |  | NA / Y / PY / PN / N / NI |
| **2.5. If Y/PY/NI to 2.4: Were these deviations from intended intervention balanced between groups?** |  | NA / Y / PY / PN / N / NI |
| **2.6 Was an appropriate analysis used to estimate the effect of assignment to intervention?** | An intention to treat analysis was used to estimate effect of the intervention. | Y / PY / PN / N / NI |
| **2.7 If N/PN/NI to 2.6: Was there potential for a substantial impact (on the result) of the failure to analyse participants in the group to which they were randomized?** |  | NA / Y / PY / PN / N / NI |
| **Risk-of-bias judgement** | Risk of bias from lack of allocation concealment creates a high risk of bias overall. | Low / High / Some concerns |
| Optional: What is the predicted direction of bias due to deviations from intended interventions? |  | NA / Favours experimental / Favours comparator / Towards null /Away from null / Unpredictable |

Domain 2: Risk of bias due to deviations from the intended interventions (*effect of adhering to intervention*)

| **Signalling questions** | **Comments** | **Response options** |
| --- | --- | --- |
| **2.1. Were participants aware of their assigned intervention during the trial?** |  | Y / PY / PN / N / NI |
| **2.2. Were carers and people delivering the interventions aware of participants' assigned intervention during the trial?** |  | Y / PY / PN / N / NI |
| **2.3. [If applicable:] If Y/PY/NI to 2.1 or 2.2: Were important non-protocol interventions balanced across intervention groups?** |  | NA / Y / PY / PN / N / NI |
| **2.4. [If applicable:] Were there failures in implementing the intervention that could have affected the outcome?** |  | NA / Y / PY / PN / N / NI |
| **2.5. [If applicable:] Was there non-adherence to the assigned intervention regimen that could have affected participants’ outcomes?** |  | NA / Y / PY / PN / N / NI |
| **2.6. If N/PN/NI to 2.3, or Y/PY/NI to 2.4 or 2.5: Was an appropriate analysis used to estimate the effect of adhering to the intervention?** |  | NA / Y / PY / PN / N / NI |
| **Risk-of-bias judgement** |  | Low / High / Some concerns |
| Optional: What is the predicted direction of bias due to deviations from intended interventions? |  | NA / Favours experimental / Favours comparator / Towards null /Away from null / Unpredictable |

Domain 3: Missing outcome data

| **Signalling questions** | **Comments** | **Response options** |
| --- | --- | --- |
| **3.1 Were data for this outcome available for all, or nearly all, participants randomized?** | All of the participants data was collected for outcomes assessed immediately after the endoscopic procedure and for the duration that the participant remained in a healthcare setting post-operatively. At the 12 month follow-up, nearly all of the participants’ data was available, in the event of the data not being available, the numbers and the reasons for this were disclosed such as discontinued intervention or death. | Y / PY / PN / N / NI |
| **3.2 If N/PN/NI to 3.1: Is there evidence that the result was not biased by missing outcome data?** |  | NA / Y / PY / PN / N |
| **3.3 If N/PN to 3.2: Could missingness in the outcome depend on its true value?** |  | NA / Y / PY / PN / N / NI |
| **3.4 If Y/PY/NI to 3.3: Is it likely that missingness in the outcome depended on its true value?** |  | NA / Y / PY / PN / N / NI |
| **Risk-of-bias judgement** |  | Low / High / Some concerns |
| Optional: What is the predicted direction of bias due to missing outcome data? |  | NA / Favours experimental / Favours comparator / Towards null /Away from null / Unpredictable |

Domain 4: Risk of bias in measurement of the outcome

| **Signalling questions** | **Comments** | **Response options** |
| --- | --- | --- |
| **4.1 Was the method of measuring the outcome inappropriate?** | No, the methods used to measure primary and secondary outcomes were appropriate, with the primary outcome assessing the number of endoscopic dilations needed post-treatment, complications were also assessed appropriately according to clinical presentation. | Y / PY / PN / N / NI |
| **4.2 Could measurement or ascertainment of the outcome have differed between intervention groups?** | Np, the measurement of outcomes was the same for both groups in the randomised trial. | Y / PY / PN / N / NI |
| **4.3 If N/PN/NI to 4.1 and 4.2: Were outcome assessors aware of the intervention received by study participants?** |  | NA / Y / PY / PN / N / NI |
| **4.4 If Y/PY/NI to 4.3: Could assessment of the outcome have been influenced by knowledge of intervention received?** |  | NA / Y / PY / PN / N / NI |
| **4.5 If Y/PY/NI to 4.4:** **Is it likely that assessment of the outcome was influenced by knowledge of intervention received?** |  | NA / Y / PY / PN / N / NI |
| **Risk-of-bias judgement** |  | Low / High / Some concerns |
| Optional: What is the predicted direction of bias in measurement of the outcome? |  | NA / Favours experimental / Favours comparator / Towards null /Away from null / Unpredictable |

Domain 5: Risk of bias in selection of the reported result

| **Signalling questions** | **Comments** | **Response options** |
| --- | --- | --- |
| **5.1 Were the data that produced this result analysed in accordance with a pre-specified analysis plan that was finalized before unblinded outcome data were available for analysis?** | Yes, the study had pre-determined study end points, addressing which outcomes would be measured, how they would be measured and at what time point. Statistical analysis was also detailed, specifying the methods, calculations and statistical tests that would be used. | Y / PY / PN / N / NI |
| **Is the numerical result being assessed likely to have been selected, on the basis of the results, from...** |  |  |
| **5.2. ... multiple eligible outcome measurements (e.g. scales, definitions, time points) within the outcome domain?** | The same scales and definitions for the outcome measurements were maintained throughout the RCT. There were multiple time points used to assess outcomes, however this is justified as it allowed follow-up of the participants to assess the clinical effectiveness of the intervention over time. | Y / PY / PN / N / NI |
| **5.3 ... multiple eligible analyses of the data?** | Analyses of the same data would produce the same results when using the analysis techniques that were detailed in the RCT. | Y / PY / PN / N / NI |
| **Risk-of-bias judgement** |  | Low / High / Some concerns |
| Optional: What is the predicted direction of bias due to selection of the reported result? |  | NA / Favours experimental / Favours comparator / Towards null /Away from null / Unpredictable |

Overall risk of bias

| **Risk-of-bias judgement** | In most domains, the overall risk of bias was low, however the inability to blind participants from their assigned intervention does introduce concerns regarding performance and detection bias. | Low / High / Some concerns |
| --- | --- | --- |
| Optional: What is the overall predicted direction of bias for this outcome? |  | NA / Favours experimental / Favours comparator / Towards null /Away from null / Unpredictable |


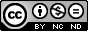


This work is licensed under a [Creative Commons Attribution-NonCommercial-NoDerivatives 4.0 International License](http://creativecommons.org/licenses/by-nc-nd/4.0/).
