# Supplementary figures and images for: SX-ELLA biodegradable stent for benign oesophageal strictures: a systematic review and proportion meta-analysis
Source: Surg Endosc. 2022 Dec 8;37(4):2476–84. doi: 10.1007/s00464-022-09767-w (PMC10082093; doi:10.1007/s00464-022-09767-w)

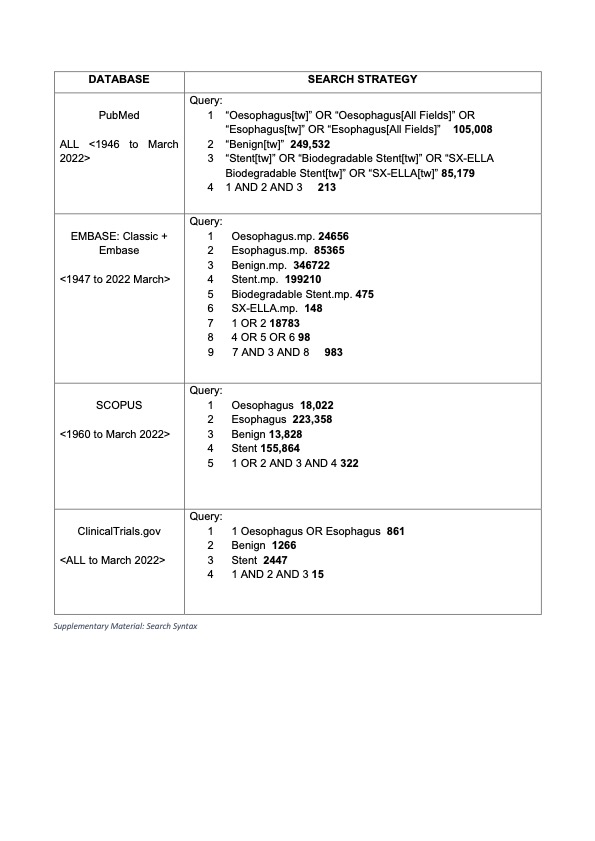

Supplement: Supplementary file 17 — Supplementary file17 (JPG 70 KB) [file 464_2022_9767_MOESM17_ESM.jpg]
